# Lipidome alteration as a hallmark and therapeutic target in Alzheimer’s disease

**DOI:** 10.4103/NRR.NRR-D-25-00709

**Published:** 2026-01-27

**Authors:** Sijia He, Xianlin Han

**Affiliations:** Barshop Institute for Longevity and Aging Studies, University of Texas Health San Antonio, San Antonio, TX, USA; Department of Cellular and Integrative Physiology, University of Texas Health San Antonio, San Antonio, TX, USA; Division of Diabetes, Department of Medicine, University of Texas Health San Antonio, San Antonio, TX, USA

Alzheimer’s disease (AD) is a progressive neurodegenerative disorder marked by cognitive decline and memory loss. Its well-established pathological features include the presence of extracellular amyloid-beta (Aβ) plaques, intracellular tau-containing neurofibrillary tangles, and neuroinflammation (He et al., 2025). While AD research over the past few decades has focused mainly on genetic and protein-centric mechanisms, a growing body of evidence points to lipids as a critical, yet previously underappreciated dimension of AD pathology. Recent advances in lipidomics emphasize that lipid dysregulation is not merely a secondary disease phenomenon, but rather a central component, and potentially a driving force of AD progression. This perspective article highlights altered lipid metabolism as both a hallmark and a promising therapeutic target for AD.

**Lipidome — more than a structural footnote:** Lipids are a critical component of the brain tissue, notable for their high abundance and structural diversity. They constitute over 50% of dry weight of the brain and perform a wide range of functions in the central nervous system beyond serving as membrane constituents. These include involvement in signal transduction, synaptic plasticity, neuroinflammation, and myelination. Several unique lipid classes and molecular species, such as long-chain polyunsaturated fatty acids, including arachidonic acid, eicosapentaenoic acid, and docosahexaenoic acid, are considered crucial for neuronal health. The need to understand the dynamics of lipid composition as well as function under various physiological/pathological conditions has significantly driven the development of lipid detection and quantification techniques for the last two decades (Han and Gross, 2003). Recent development of techniques in sample preparation, microsampling methods, lipidomics imaging, single-cell lipidomics, and lipidomics data analysis greatly enhanced the capacity and sensitivity for lipid detection. Particularly, mass spectrometry-based lipidomics emerged as a powerful approach for profiling lipid species in high resolution and identifying lipid spatial distribution (He et al., 2025). These lipidomics developments greatly deepened our understanding of lipid diversity and function in the central nervous system. In the context of AD, lipidomics has revealed distinct alterations in the brain lipidome, highlighting changes across multiple lipid classes. A major category of lipids that exhibit an AD pattern is sphingolipids that is highly enriched in the central nervous system and particularly important for myelin function. Among which, elevated ceramide levels are often observed in the AD brain compared to normal individuals, while other ceremide-related lipids exhibit brain region-, and disease stage-specific changes in AD. For example, sphingomyelin is decreased in the middle frontal gyrus but elevated in the neocortex, entorhinal cortex, and grey matter; ganglioside is down-regulated in the hippocampus and prefrontal cortex but induced in the entorhinal cortex; up-regulated sphingosine has been detected in multiple regions with pathology. Mechanistically, sphingolipids dysregulation can modulate the progression of AD by influencing Aβ, tau, neuroinflammation, and via apolipoprotein E (ApoE) function. For example, sulfatides (ST, a major group of myelin-enriched glycosphingolipids), are consistently down-regulated in various brain regions in AD (Xu et al., 2024). Importantly, this downregulation appears to occur early in the disease course and has been found to be mediated by ApoE (Han et al., 2002), indicating that ST depletion may serve as a specific and sensitive marker of AD-specific lipid alteration. Supporting this notion, follow-up studies using mouse models demonstrated that ST loss induces neuroinflammation and leads to AD-like phenotypes, including both central cognitive decline and peripheral deficits (Qiu et al., 2021, 2023; He et al., 2023). These findings, together with previous studies of other lipid metabolism genes (e.g., ABCA7 and triggering receptor expressed on myeloid cells 2 [Trem2]) that showed impact on AD risk (Wang et al., 2015; Kawatani et al., 2024), further underscore the pathogenic role of lipid dysregulation in driving AD progression. In addition to sphingolipids, other lipid classes are also known to exhibit a clear pattern in human AD brain, including the downregulation of phospholipids and their derivatives, upregulation of glycerolipids, and the elevation of cholesterol (He et al., 2025).

**Mechanistic links between lipid dysregulation and Alzheimer’s disease pathogenesis:** Supporting the notion that lipid alterations serve as a hallmark of AD, there are multiple layers of evidence demonstrating the mechanistic connection of lipidome changes with established AD features. At the genomic level, it is well recognized that ApoE, a major lipid transporter in the brain, stands out as the strongest genetic risk factor for AD. Carriers of the ApoE4 isoform have a markedly increased risk of developing the disease, while the ApoE2 isoform confers protection. It was reported that poor lipidation of APOE4 prevents efficient transport of lipids between glia and neurons, which contributes to AD risk. ApoE has been shown to serve as a carrier for ST, potentially contributing to the observed ST depletion in AD. This is supported by the observation that mice carrying the ApoE4 isoform has the lowest ST levels in the brain compared to ApoE2 or ApoE3 (Han, 2010). Given essential role of ApoE in Aβ clearance, the ST-ApoE complex may be a critical modulator of Aβ metabolism. Another notable AD risk gene is the Trem2, a microglia-enriched cell surface receptor for various ligands, including lipids (such as ST, phosphatidylserine, and lipoproteins). Trem2 facilitates microglial clearance of Aβ and participates in tau phosphorylation, seeding, and propagation. The lipid sensing function of Trem2 has been reported to sustain microglial response, the R47H variant of TREM2, which impairs lipid sensing, is strongly associated with elevated AD risk (Wang et al., 2015). Beyond ApoE and Trem2, recent genome-wide association studies have revealed a broader spectrum of lipid-related genes influencing AD susceptibility, these include: lipid transport-related genes such as *SORL1*, *ABCA1*, and *ABCA7*; genes that encode lipid modification factors such as *INPP5D* and *PLCG2*; genes that directly regulate lipid metabolism — *PRKD3* and *KLF16*; as well as those play indirect roles such as *ADAM17*, *HS3ST5*, *FERMT2*, and *ADAMTS1* (He et al., 2025).

Another aspect of lipid-associated AD mechanism is based on their central contribution to Aβ pathology. Early descriptions of amyloid as “waxy” and “lardaceous” hinted at its lipid-rich nature, and this observation is now supported by biochemical analyses showing that amyloid plaques contain significant lipid content, including cholesterol, sphingomyelin, ST, and ceramides. Advances in lipidomics have revealed that specific lipid species, such as lysophospholipids and bis(monoacylglycero)phosphates, accumulate around Aβ plaques in both human AD brains and mouse models, highlighting the complex interplay between lipids and Aβ metabolism. Lipids regulate multiple stages of Aβ biology, including APP processing, Aβ aggregation, and Aβ clearance. Lipid raft composition, especially enriched in cholesterol and sphingolipids, influences β- and γ-secretase activities, while altered membrane lipid profiles (e.g., reduced polyunsaturated fatty acids and elevated ceramides) enhance Aβ production. Exosomes, enriched in specific lipids, may mediate Aβ secretion and aggregation. ST has emerged as a regulator of Aβ clearance through ApoE, and ST supplementation inhibits Aβ production by suppressing secretase activity *in vitro*. Furthermore, Aβ-lipid interactions contribute directly to toxicity by disrupting membrane integrity, altering calcium signaling, and promoting oxidative stress (He et al., 2025).

Increasing evidence also highlights the critical role of lipids in modulating tau pathophysiology. Paired helical filament tau associates with lipid-rich structures such as the endoplasmic reticulum and membrane microdomains. Glycolipid, phosphatidylcholine, galactocerebrosides, sphingomyelin, and cholesterol were found to be included in paired helical filament samples. Negatively charged phospholipids interact electrostatically with tau’s positively charged domains, promoting conformational changes and aggregation. Altered lipid metabolism also drives tau hyperphosphorylation by activating kinases (e.g., glycogen synthase kinase 3β and cyclin–dependent kinase 5) via pathways sensitive to phosphoinositide levels, lipid raft integrity, and cholesterol homeostasis. Furthermore, lipid interactions facilitate tau secretion and propagation, including direct membrane translocation aided by cholesterol, sphingolipids, PI(4,5)P₂, and extracellular heparan sulfate proteoglycans. These findings underscore lipidomic dysregulation as a critical contributor to tau pathology.

Neuroinflammation, driven by sustained activation of glial cells such as microglia and astrocytes, is a key contributor to AD progression. Mountains of evidence suggest that lipids play central roles in modulating neuroinflammatory responses. Dysregulated lipid classes, including cholesterol, oxysterols, and ST, can promote microglial activation, oxidative stress, and proinflammatory cytokine release. Lipid droplet-accumulating microglia, marked by impaired phagocytosis and elevated reactive oxygen species, are prevalent in aging and AD brains. Lipid derivatives such as 27-hydroxycholesterol and 7-ketocholesterol exacerbate inflammation, while others, such as 24-hydroxycholesterol, may offer neuroprotection. Conversely, anti-inflammatory lipids, including omega-3 fatty acids (docosahexaenoic acid and eicosapentaenoic acid), neuroprotectin D1, and plasmalogens, enhance Aβ clearance, suppress inflammatory mediators, and support resolution of inflammation (He et al., 2025).

In summary, the interconnection of lipids with the aforementioned aspects of AD strongly supports the emerging view of lipids as a central hallmark of the disease.

**Lipidome modification as a therapeutic strategy against Alzheimer’s disease:** Current therapeutic strategies for AD largely focus on symptom management through behavioral interventions and pharmacological treatments. While drugs such as acetylcholinesterase inhibitors and N-methyl-D-aspartate receptor antagonists aim to alleviate cognitive decline, newer U.S. Food and Drug Administration-approved therapies such as aducanumab, lecanemab, and donanemab target Aβ plaque accumulation. Despite the growing recognition that lipid metabolic dysfunction plays a key role in AD pathogenesis, lipid-targeted therapeutics remain underexplored. Recent efforts suggest that modulating lipid metabolism through synthesis, efflux, storage, or modification holds potential for disease prevention. Moreover, dietary interventions offer an accessible avenue for influencing brain lipid composition and AD progression.

Targeting cholesterol synthesis with statins, although effective in cardiovascular disease, has yielded mixed results in AD, potentially due to the dual role of membrane cholesterol in both APP processing and neuronal function, or other unknown factors. Enhancing lipid efflux via ABCA1 activation or ApoE2 gene therapy has shown promise in reducing Aβ and tau pathologies in preclinical models. Nuclear receptors such as LXRs, which regulate cholesterol metabolism and inflammation, offer another therapeutic avenue, though avoiding side effects would be necessary for clinical success. In terms of lipid storage, inhibiting enzymes such as ACAT1 may reduce harmful cholesterol esters and promote neuroprotection. Similarly, activation of PPARγ has shown anti-inflammatory and cognitive benefits, although clinical outcomes remain inconsistent.

Lipid modification pathways, particularly those involving phospholipases and oxidative lipid damage, have emerged as novel targets. Inhibiting specific PLA2 isoforms may reduce neuroinflammation and Aβ toxicity, while restoring protective lipids such as plasmalogens or modulating fatty acid desaturation may improve cognitive outcomes. Intervention with nervonic acid has the potential to be developed as an antioxidant drug for the prevention and early therapy of AD. Lastly, dietary supplementation of omega-3 polyunsaturated fatty acids, known for their anti-inflammatory and neuroprotective properties, have demonstrated variable clinical efficacy, highlighting the need to consider the genetic background, disease stage, and combination therapies. Together, these findings underscore the therapeutic potential of targeting lipid metabolism as a multifaceted strategy in AD treatment (He et al., 2025).

**Conclusion:** Emerging evidence from lipidomics research has transformed our understanding of AD, positioning lipid dysregulation as both a hallmark and a mechanistic driver of its pathogenesis. Far beyond their traditional roles as structural components, lipids actively shape neuronal function, protein aggregation, neuroinflammation, and intercellular signaling. Alterations in specific lipid classes, particularly sphingolipids, phospholipids, and cholesterol, are now recognized as mechanistically relevant features of AD. Genetic insights further support this view, with several AD-associated risk genes intricately linked to lipid metabolism and transport.

These findings underscore a paradigm shift in AD research: from a protein-centric to a more integrative model that embraces lipid biology as central to disease initiation and progression. Importantly, they also open new avenues for therapeutic innovation. Modulating lipid metabolism through pharmaceutical, genetic, or dietary means offers a promising strategy to influence multiple aspects of AD pathology simultaneously, from amyloid accumulation and tau aggregation to neuroinflammation and synaptic dysfunction.

**Figure 1:**
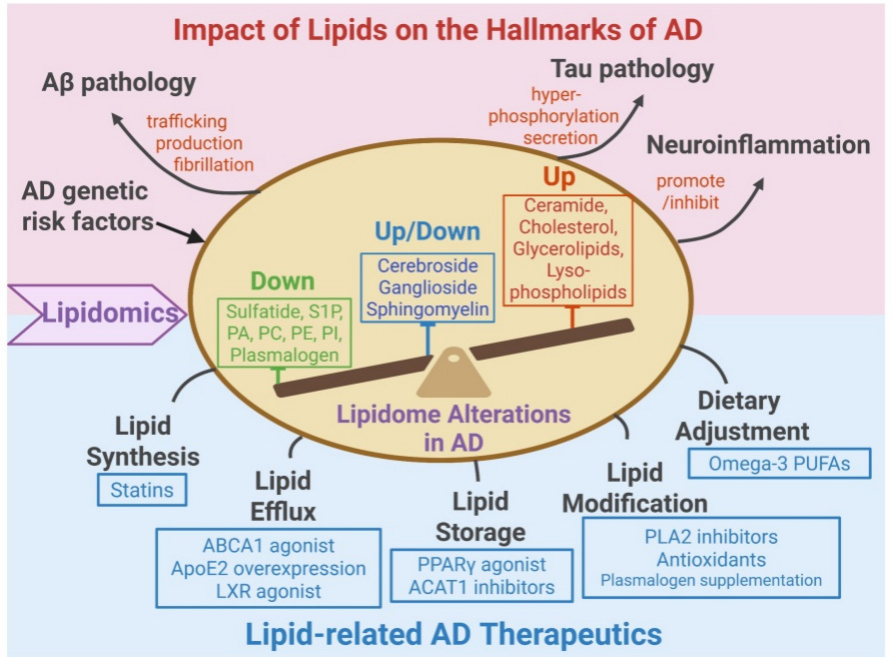
Lipidome alterations are both a hallmark and a potential therapeutic target of AD. Advances in lipidomics have significantly facilitated the characterization of AD-specific lipidomic profiles. These alterations are mechanistically linked to the disease process by interacting with key pathological features of AD, including genetic mutations, Aβ pathology, tau pathology, and neuroinflammation. Furthermore, several promising therapeutic strategies have been proposed by targeting lipid metabolism, including lipid synthesis, efflux, storage, and modification. Created in BioRender. He, S. (2025) https://BioRender.com/6uuoflt. Aβ: Amyloid-beta; AD: Alzheimer’s disease; ABCA1: ATP binding cassette subfamily A member 1; ACAT: Acyl-CoA cholesterol acyltransferase; ApoE2: apolipoprotein E2; LXR: liver X receptor; PA: phosphatidic acid; PC: phosphatidylcholine; PE: phosphatidylethanolamine; PI: phosphatidylinositol; PLA2: phospholipase A2; PPARγ: peroxisome proliferator-activated receptor gamma; PUFA: polyunsaturated fatty acids.

As the field advances, future research should focus on several key areas to fully harness the potential of lipidomics in AD: (1) clarify the causal relationships between lipid alterations and AD progression (e.g., decipher lipid-Aβ-tau interactions through multi-omics integration); (2) identify lipid-based biomarkers for early diagnosis and develop personalized lipid-modifying therapies that tailored to individual genetic backgrounds (e.g., sporadic versus familial AD) and disease stages; (3) address methodological gaps to refine lipid-centric diagnostic and intervention strategies; (4) leverage precision delivery methods (e.g., nanoparticle carriers) to mitigate off-target risks of treatment; (5) integrate AI-driven approaches to accelerate discovery and translation in this domain. Ultimately, embedding lipidomics into mainstream AD research and clinical practice may provide a powerful lever to modify disease trajectory and improve patient outcomes (**Figure 1**).

*This work was supported by National Institute on Aging grants R01AG061872 (to XH), RF1AG061729 (to XH), R01AG085545 (to XH), T32AG021890 (to SH), P30AG013319, and P30AG044271, the Methodist Hospital Foundation (to XH), the Cure Alzheimer’s Fund (to XH), the Owen Foundation (to XH), the American Federation for Aging Research (to SH), and the Owen Foundation (to SH).*
